# Long-Lasting Insecticidal Net Ownership and Use in Mogode Health District, Cameroon

**DOI:** 10.7759/cureus.57819

**Published:** 2024-04-08

**Authors:** Collins Buh Nkum, Jerome Ateudjieu, Aude Nanfak, Ketina Hirma Tchio-Nighie, Sonia Imelda Mbiaketcha Nzinnou, Etienne Guenou, Landry Beyala Bita'a, Willy Armand Nguemnang Nguemnang, Charlette Nangue, Georges Nguefack-Tsague

**Affiliations:** 1 Department of Health Research, M.A. SANTE (Meilleur Accès aux Soins de Santé), Yaounde, CMR; 2 Department of Public Health, Faculty of Medicine and Biomedical Sciences, University of Yaounde 1, Yaounde, CMR; 3 Department of Public Health, Faculty of Medicine and Pharmaceutical Sciences, University of Dschang, Dschang, CMR; 4 Division of Health Operations Research, Ministry of Public Health, Yaounde, CMR

**Keywords:** household, mogode health district, llin use, ownership, long-lasting insecticide-treated net

## Abstract

Background: Cameroon is a malaria-endemic country. Many control strategies including long-lasting insecticidal nets (LLIN) have been proposed to reduce the burden of malaria. The World Health Organization aims to achieve at least 80% of people sleeping under a LLIN. This study assessed the ownership and use of LLNs in the Mogode Health District (MHD).

Methods: A community-based cross-sectional study was conducted in MHD in September 2021. Data on ownership and LLINs use were collected using structured questionnaires following the Roll Back Malaria guidelines. Univariate and multivariate analyses were performed to assess the determinants of ownership and failure to LLIN use.

Results: A total of 332 households were included from eight health areas. The proportion of households with at least one LLIN was 72.0% (238). However, 232 (70.0%) reported having used LLIN (sleeping under LLIN the previous night). Household heads with higher education were six times more likely to have owned LLINs than those with no education (adjusted odds ratio (AOR)=6.8; confidence interval (CI) 1.5, 31.0, p< 0.05). Additionally, household heads between the ages of 36-50 were 4.2 times (AOR= 4.2, CI 1.3-13.8, p< 0.05) likely to fail to use LLINs in households. However, households where heads had secondary education (AOR= 0.2, CI 0.1-0.6, p< 0.05), were negatively associated with failure to use LLINs.

Conclusion: Ownership and use of LLINs in MHD remain challenging. Therefore, this finding will contribute to improving recommendations and updating strategies such as targeted messages aimed at raising awareness of malaria during mass LLIN distribution campaigns.

## Introduction

Malaria is one of the most common parasitic diseases in the world [1]. If left untreated, malaria-infected patients may develop severe complications and lead to death., Many control strategies have been put in place to reduce the burden of malaria such as long-lasting insecticidal nets (LLIN), indoor residual spraying (IRS), accurate diagnosis, prompt treatment with artemisinin-based combination therapies (ACTs), and intermittent preventive treatment of pregnant women (IPTp) [2,3]. However, despite all these strategies, the burden remains high. In 2020, an estimated 241 million cases of malaria occurred worldwide, with 627,000 deaths and 95% reported in Africa [4]. In 2021, the World Health Organization (WHO) reported more than six million cases and more than 13,000 deaths due to malaria in Cameroon [5]. Malaria accounts for the greatest proportion of hospitalizations (56%) and deaths (26%) in the far north region of Cameroon [6].

In recent years, LLINs have become the preferred tool for malaria control and are known to be highly beneficial in reducing the malaria burden [7-10]. To reach and sustain universal coverage of LLINs, the WHO recommends that countries use a combined approach of mass distribution of free nets through campaigns and continuous distribution through multiple channels, particularly through antenatal clinics (ANC) and the expanded program on immunization (EPI) [11]. Mass campaigns are the only cost-effective and proven way to achieve high and equal coverage in a timely manner [11]. However, achieving universal LLIN coverage and use remains a major challenge for Africa. In 2018, 72% of households had access to a LLIN, but only 40% of the population slept under one [3].

In Cameroon, the mass distribution campaign of LLINs was first introduced in 2011, with approximately eight million LLINs distributed [12]. This was followed by a second mass distribution in 2015 and a third with a distribution of approximately eight million LLINs in 2019 [2]. From 2011 to 2018, surveys increased in LLIN ownership (36%-73%) and usage (15%-54%) [13].

Demographics, socioeconomic status, and household size have been identified as factors influencing the ownership and usage of LLINs in northwest and southwest Cameroon [14,15]. To assess the real benefits and effects of LLINs on malaria prevalence, ownership alone is not an effective indicator because use is a key expectation. Unfortunately, few studies have been carried out to assess the ownership and use of LLINs in Cameroon after various distribution campaigns [7,14,16,17]. In order to contribute to this assessment, this study was designed to assess LLIN ownership and use in the Mogode Health District (MHD).

## Materials and methods

Study design 

A community-based cross-sectional descriptive study was conducted in September 2021 targeting household heads to assess the ownership and use of LLINs in the MHD population. Data were collected from questionnaires administered to household heads face-to-face by community health workers (CHWs).

Study area

**Figure 1:**
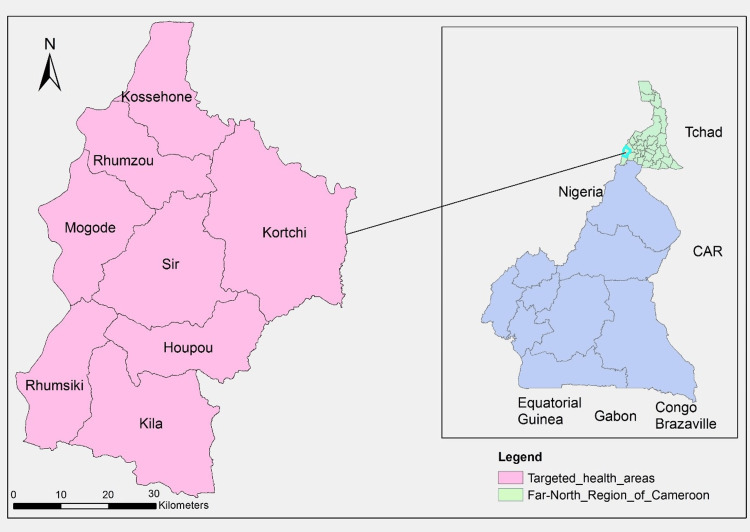
Map of the Mogode Health District

The study was implemented in the MHD in the far north region of Cameroon. The MHD is one of the 30 health districts of the Far North region of Cameroon having a population of 137,283 in 2020. It is composed of eight health areas and 14 healthcare facilities (Figure 1). It shares boundaries with the Adamawa state of Nigeria and three health districts in Cameroon, including Hina, Mokolo, and Bourrha. It is a point of a very high population exchange between Nigeria and Cameroon with many refugees fleeing from the Boko Haram crossing to go to the Minawao refugee camp. This situation makes the population face difficulties regarding access to care.

Study population

The study targets were all selected households in the MHD. Household respondents were people aged 18 years or older who had been living in the health district for more than one year. 

Sample size determination

The minimum needed sample size for the study was 315 households, assuming that 75.4% of households owned at least one LLIN [18], with a precision of 5%, a significance level of 5%, a power of 95%, and a nonresponse rate of 10%. The sample was distributed to different health areas with respect to population size.

Sampling

All health areas of the MHD were targeted. Households in each health area were randomly listed and consecutively visited following a randomly assigned order.

Data collection procedure

Data were collected by community health workers fluent in the local language. In each selected household, the study objectives and procedures were presented to the head of the household, who was then invited to participate in the study. The health workers used the study information notice. We included households with respondents aged above 18 years or older who had been living in the health district for more than one year. Households where the surveyor was unable to meet the head of household or his/her representative after two visits during the survey period were excluded. Those willing to participate signed the consent form. After obtaining informed consent, a questionnaire was administered to collect the sociodemographic characteristics of respondents, LLIN ownership, and use. In the context of the present study, LLIN ownership is defined as having at least one LLIN in the household, and LLIN use is defined as sleeping under LLIN the previous night.

Ethical consideration

Ethical clearance was obtained from the University of Yaounde Institutional Review Board (ethical clearance No 638/UY1/FMSB/VDRC/DAASR/CSP). We also obtained research authorizations from the MHD. Prior to the administration of each questionnaire, the surveyor fully explained to each respondent on the basis of an information sheet the objectives and procedures of the study, and the written consent of those willing to participate was collected.

Data processing and analysis

Data were sent to the remote server of the Kobo platform every evening during the collection period for cleaning and identifying inconsistencies. At the end of data collection, data were downloaded into an Excel database on which statistical analysis was performed using Statistical Package for Social Science (SPSS) version 17.0 (IBM Corp., Armonk, NY). Univariate and multivariate logistic regression were performed to assess determinants of ownership and failure to LLIN use. The association with the following variables was assessed: demographic characteristics of the household head (age, gender, education level, occupation, marital status, and religion), family size, and number of children below five years. A 95% confidence interval (CI) was calculated and values of P < 0.05 were considered statistically significant.

## Results

Sociodemographic characteristics of the study population

A total of 332 households were surveyed; 156 (47.0%) respondents were women, 136 (41.0%) had secondary education and 66 (19.9%) were traders. Two hundred and eight respondents (65.7%) were married, 178 (53.6%) were Muslim, and 154 (46.4%) were Christian. Furthermore, the majority (196, 59.0%) of the households surveyed had a family size of four to nine persons in the household, and 306 (92. 2%) had zero to three children (Table 1).

**Table 1:** Sociodemographic characteristics of the study population

Characteristics		n (332)	%
Gender	Female	156	47.0
	Male	176	53.0
Age of household head	15-25	66	19,9
	26-35	116	34,9
	36-50	84	25,3
	˃ 50	66	19,9
Education level	None	48	14.5
	Primary	92	27.7
	Secondary	136	41.0
	Higher	56	16.9
Occupation	Farmer	34	10.2
	Trader	66	19.9
	Student	54	16.3
	Housewife	82	24.7
	Government employee	38	11.4
	Other	58	17.5
Marital status	Single	76	22.9
	Married/ Concubinage	208	65.7
	Divorced/Widowed	38	11.4
Religion	Christian	154	46.4
	Muslim	178	53.6
Family size	1-3	42	12.7
	4-9	196	59.0
	˃9	94	28.3
Number of children below 5 years	0-3	306	92.2
	4-8	26	7.8

LLIN ownership and utilization within households

Of the 332 households surveyed, 238 (72.0%) owned at least one LLIN and 232 (70.0%) used LLINs.

Factors associated with LLIN ownership 

Households whose heads had higher education were six times more likely to have owned LLINs than those whose heads had no education (adjusted odds ratio (AOR) = 6.8; 95% CI 1.5, 31.0). Households with respondents who were government employees were five times more likely to have owned LLINs than those who were farmers (AOR = 5.0; 95% CI 0.9, 27.6). Households whose heads were Muslims were less likely to have owned LLINs than Christians (AOR = 0.7; 95% CI 0.4, 1.3) (Table 2).

**Table 2:** Univariate and multivariate risk factor analysis for LLIN ownership in MHD (N = 332) LLIN: Long-Lasting Insecticidal Net: * p < 0.05; ** p < 0.001.

Covariates		LLINs Ownership					Univariate		Multivariate	
		n	N	(%)	95% CI	p-value	OR	95% CI	AOR	95% CI
Gender	Female	118	156	75.6	(68.4;81.8)	0.13	1			
	Male	120	176	68.1	(61;74.7)		0.7	(0.4; 1.1)		
Age of household head	15-25	56	66	84.8	(74.7;91.9)	0.000^*^	1		1	
	26-35	90	116	77.5	(69.3;84.4)		0.7	(0.3;1.3)	0.3	(0.09;0.9)*
	36-50	56	84	66.6	(56.1;76)		0.4	(0.1;0.8)*	0.2	(0.04;0.5)*
	˃ 50	36	66	54.5	(42.5;66.1)		0.2	(0.09;0.4)**	0.2	(0.05;0.6)*
Education level	None	24	48	50	(36.2;63.7)	0.000^*^	1		1	
	Primary	64	92	69.5	(59.6;78.2)		2.3	(1.1;4.6)*	2.1	(0.9;4.6)
	Secondary	98	136	72	(64.1;79)		2.6	(1.3;5.1)*	1.7	(0.74;3.9)
	Higher	52	56	92.8	(83.9;97.5)		13	(4.1;41.6)**	6.8	(1.5;31.0)*
Occupation	Farmer	20	34	58.8	(42.1;74)	0.000^*^	1		1	
	Trader	38	66	57.5	(45.5;68.9)		1.0	(0.4;2.2)	0.9	(0.35;2.08)
	Student	44	54	81.4	(69.6;90)		3.1	(1.1;8.1)*	0.4	(0.1;1.8)
	Housewife	62	82	75.6	(65.5;83.9)		2.2	(0.9;5.1)	1.7	(0.6;4.1)
	gouvernement employee	36	38	94.7	(84.1;98.8)		12.6	(2.6;61.1)*	5.0	(0.9;27.6)
	Other	38	58	65.5	(52.7;76.7)		1.3	(0.5; 3.1)	1.0	(0.4;2.5)
Marital status	Single	60	76	78.9	(68.8;86.9)	0.18	1			
	Married/Concubinage	154	218	70.6	(64.3;76.3)		0.6	(0.3;1.2)		
	Divorced/Widowed	24	38	63.1	(47.3;77)		0.5	(0.19;1.1)		
Religion	Christian	124	154	80.5	(73.7;86.1)	0.001	1		1	
	Muslim	114	178	64	(56.8;70.8)		0.4	(0.2;0.7)*	0.7	(0.4;1.3)
Family size	1-3	28	42	66.6	(51.6;79.4)	0.06	1			
	4-9	150	196	76.5	(70.2;82)		1.6	(0.8;3.3)		
	˃9	60	94	63.8	(53.8;73)		0.9	(0.4;1.9)		
Number of children below 5 years	0-3	216	306	70.5	(65.3;75.4)	0.13	1			
	4-8	22	26	84.6	(67.4;94.5)		2.3	(0.7;6.8)		

Factors associated with LLIN use in households

Gender, age of the household head, education level, professional activity, and household size remained independent risk factors for failure to use LLINs in the household in the multivariate model. Failure to use LLINs was more likely in households with male heads AOR = 1.6; 95% CI 0.8, 3.4), households in which the heads were between 36 and 50 years old (AOR = 4.2; 95% CI: 1.3, 13.8) and above 50 years old (AOR = 3.9; 95% CI: 1.2, 12.8) were more likely to fail to use LLIN than those in which the heads were under 35 years old. Failure to use LLIN was less common in households where heads had a secondary education level (AOR = 0.2, 95% CI: 0.1, 0.6), and who were government employees (AOR = 0.2; 95% CI: 0.1, 1.1), compared to households where heads had no education level and were farmers, respectively. In addition, households with large families (˃9 members) had higher odds of failure to use LLINs (AOR = 2.2; 95% CI: 0.7, 7.0) than households with smaller families (Table 3).

**Table 3:** Univariate and multivariate risk factor analysis for failure to use LLINs in households of MHD (N = 332) LLIN: Long-Lasting Insecticidal Net; * p < 0.05; ** p < 0.001.

Covariates		Failure for using LLINs					Univariate		Multivariate	
		n	N	%	95% CI	p	OR	95% CI	AOR	95% CI
Gender	Female	32	156	20.5	(14.7; 27.3)	0.000^*^	1		1	
	Male	68	176	38.6	(31.6; 45.9)		2.4	(1.5, 4)^**^	1.6	(0.8, 3.4)
Age of household head	15-25	18	66	27.3	(17.6; 38.8)	0.000^*^	1		1	
	26-35	14	116	12.1	(7.08; 18.9)		0.4	(0.2, 0.8)^ *^	1.0	(0.3, 2.9)
	36-50	32	84	38.1	(28.2; 48.7)		1.6	(0.8, 3.3)	4.2	(1.3,13.8)^*^
	above 50	36	66	54.5	(42.5; 66.1)		3.2	(1.5, 6.6)^*^	3.9	(1.2,12.8)^*^
Education level	None	26	48	54.2	(40.1; 67.6)	0.000^*^	1		1	
	Primary	30	92	32.6	(23.6; 42.6)		0.4	(0.2, 0.8)^*^	0.4	(0.2, 0.9)^*^
	Secondary	32	136	23.5	(17; 31.1)		0.3	(0.1,0.5)^**^	0.2	(0.1, 0.6)^*^
	Higher	12	56	21.4	(12.2; 33.4)		0.2	(0.1, 0.5)^*^	0.3	(0.1, 0.9)^*^
Occupation	Farmer	18	34	52.9	(36.5; 68.9)	0.000^*^	1		1	
	Trader	20	66	30.3	(20.2; 42)		0.4	(0.2, 0.9)^ *^	0.4	(0.2, 1.2)
	Student	18	54	33.3	(21.8; 46.5)		0.4	(0.2, 1.2)	2.6	(0.6, 10.7)
	Housewife	16	82	19.5	(12; 29)		0.2	(0.1, 0.5)^ *^	0.5	(0.1, 1.6)
	Government employee	4	38	10.5	(3.65; 23.1)		0.1	(0,0.4)^ **^	0.2	(0.1, 1.1)
	Other	24	58	41.4	(29.3; 54.2)		0.6	(0.3, 1.5)	0.9	(0.3, 2.6)
Marital status	Single	22	76	28.9	(19.6;39.7)	0.96	1			
	Married/Concubinage	66	218	30.3	(24.4; 36.6)		1.1	(0.6, 1.9)		
	Divorced/Widowed	12	38	31.6	(18.5; 47.2)		1.1	(0.5, 2.6)		
Religion	Christian	34	77	44.2	(20.7; 34.6)	0.29	1			
	Muslim	52	89	58.4	(26; 39.7)		1.3	(0.8, 2.1)		
Family Size	1-3	6	42	14.3	(6.19; 27)	0.000*	1		1	
	4-9	50	196	25.5	(19.7; 31.9)		2.1	(0.8, 5.2)	1.7	(0.6, 4.8)
	Above 9	44	94	46.8	(36.9; 56.8)		5.3	(2, 13.7)^ *^	2.2	(0.7, 7.0)
Number of children below 5 years	0-3	88	306	28.8	(23.9; 34.0)	0.189	1			
	4-8	12	26	46.2	(23.9; 34.0)		2.1	(0.9, 4.8)		

## Discussion

This study assessed LLIN ownership and use in the MHD one year after the 2019/2020 mass distribution campaign.

LLIN ownership

The LLIN is an important tool for malaria control and has been adopted by many countries for malaria prevention and elimination. Our study revealed that 72% of surveyed households owned at least one LLIN, which is higher than the 47%-68.1% reported in studies conducted in Cameroon [8,14,17,19], and 62% in Uganda [20]. Conversely, a higher proportion was observed in Madagascar [21]. Similar results were obtained in Cameroon, where 75.4% of households owned at least one [18], LLIN, and in Uganda, where 73% of households owned at least one LLIN [22]. The discrepancies in results can be explained by the following reasons: different sample sizes and population densities, different study settings and designs, and some studies were hospital-based studies. In Cameroon, household ownership of LLINs increased from 65.6% to 76.6% from 2013 to 2017 and the usage rate increased from 40.4% to 58.3% from 2013 to 2017 [23]. The present study showed that one year after the campaign, 72% of households had an LLIN at the time of the survey which is below the goal of the LLIN campaign which was to reach 80% of the population sleeping under the WHO recommended [24]. The difference is 8%, highlighting the need to improve strategies to increase LLIN ownership.

Education level was among the factors affecting the ownership of LLINs in our study. This may be attributed to the significant role that education plays in the development and awareness of communities. Similar findings have been reported in other studies. In one study in southern Ethiopia, respondents with secondary or above education were more likely to own an LLIN [25]. In our study, occupation was associated with LLIN ownership, and a study conducted in Ethiopia revealed similar results [26].

LLIN use

LLIN ownership does not guarantee the expected effect of the net when acquired; it is only the use that may lead to the expected outcome of this disease response technique, and thus, to a reduction in morbidity. The proportion of household heads who slept under LLINs the previous night was 70%. Our findings were lower compared to other studies in Mezam Division (77.8%) [16]; and Fako Division (94.1%) [12], in Cameroon as well as other countries [20,21]. Similar results were reported in Buea Health District (69.7%) [17], and Bamenda Health District [14], and lower findings were reported in rural and urban Buea [19]. The discrepancy in the use of LLINs may be because different studies had different sample sizes, study designs, and times and settings (urban and rural). Households headed by married persons were more likely to fail to use LLINs than households headed by singles. Possible interpretations include greater decision-making power in parent-headed households and their role in protecting their children from disease [27]. Households with more than nine members were more likely to fail LLIN use than households with fewer members. Therefore, failure to use LLINs was correlated with family size. This was previously observed in Southwest Ethiopia [28]. This can be explained by the fact that control over a large family size is limited. The statistically significant reduction in LLIN usage in households where the heads had primary education can be explained by the poor knowledge of malaria prevention and has been reported by other studies to be associated with low levels of LLIN use [29,30]. Interventions such as information, education, and communication (IEC) and behavior change communication (BCC) should be conducted before, during, and after MDC to promote LLIN distribution, and permanent and adequate use [17]. Additional studies are needed in the northern regions of Cameroon to better understand how community perceptions and beliefs influence the sustained use of LLINs in households. Gender, age of household head, and occupation were also significantly associated with failure to use LLIN.

Limitations

The porous nature of the borders with Nigeria and the sociopolitical instability that prevails in the northern-east region of Nigeria following the terrorist activities have resulted in a state of insecurity in the border villages of Cameroon. This state of insecurity has limited our access to some villages and impacted the collection of data in some health areas of MHD.

## Conclusions

This study contributes extensively to the information on LLIN ownership and use in MHD Cameroon. LLIN ownership and utilization rates remained below the WHO goal. Gender, age of household head, education level, professional activity, and household size have an impact on LLIN use. The effectiveness of campaigns can only be guaranteed if they are followed by evaluations, in order to measure their real impact, detect their weaknesses, and above all formulate proposals that meet the expectations of both the beneficiary populations and the public authorities. This study will, therefore, contribute to formulating some recommendations for updating messages aimed at raising awareness of malaria.
